# An engineered vaccine of the *Plasmodium vivax* Duffy binding protein enhances induction of broadly neutralizing antibodies

**DOI:** 10.1038/s41598-017-13891-2

**Published:** 2017-10-23

**Authors:** Francis B. Ntumngia, Camilla V. Pires, Samantha J. Barnes, Miriam T. George, Richard Thomson-Luque, Flora S. Kano, Jessica R. S. Alves, Darya Urusova, Dhelio B. Pereira, Niraj H. Tolia, Christopher L. King, Luzia H. Carvalho, John H. Adams

**Affiliations:** 1 Center for Global Health and Infectious Diseases Research, Department of Global Health, College of Public Health, University of South Florida, Tampa, 33612 USA; 2 Centro de Pesquisas René Rachou/FIOCRUZ, Belo Horizonte, 30190 Brazil; 3 Departments of Molecular Microbiology & Microbial Pathogenesis, and Biochemistry & Molecular Biophysics, Washington University School of Medicine, Saint Louis, 63130 USA; 4 Centro de Pesquisa em Medicina Tropical de Rondonia-CEPEM, Porto Velho, 76812-245 Brazil; 5 Center for Global Health and Diseases, Case Western Reserve University, Cleveland, 44106 USA

## Abstract

*Plasmodium vivax* invasion into human reticulocytes is a complex process. The Duffy binding protein (DBP) dimerization with its cognate receptor is vital for junction formation in the invasion process. Due to its functional importance, DBP is considered a prime vaccine candidate, but variation in B-cell epitopes at the dimer interface of DBP leads to induction of strain-limited immunity. We believe that the polymorphic residues tend to divert immune responses away from functionally conserved epitopes important for receptor binding or DBP dimerization. As a proof of concept, we engineered the vaccine DEKnull to ablate the dominant Bc epitope to partially overcome strain-specific immune antibody responses. Additional surface engineering on the next generation immunogen, DEKnull-2, provides an immunogenicity breakthrough to conserved protective epitopes. DEKnull-2 elicits a stronger broadly neutralizing response and reactivity with long-term persistent antibody responses of acquired natural immunity. By using novel engineered DBP immunogens, we validate that the prime targets of protective immunity are conformational epitopes at the dimer interface. These successful results indicate a potential approach that can be used generally to improve efficacy of other malaria vaccine candidates.

## Introduction

Malaria is a major global public health problem and is associated with the lack of social and economic development of vast areas of tropical and sub-tropical countries. More people are at risk worldwide from *Plasmodium vivax* than *P*. *falciparum* ^1,2^. Despite its wide prevalence, *P*. *vivax* malaria has received little attention over the years compared to *P*. *falciparum* malaria, which is responsible for most malaria-attributed deaths. Increasing evidence of drug-resistant *P*. *vivax* strains, the evolution of more virulent forms of the parasite with associated morbidity and mortality, as well as the formation of hypnozoites with the potential for relapse^3–7^ are a cause for concern. Therefore, there is a need to develop a vaccine to control the disease caused by *P*. *vivax*.

Like all malaria parasites, merozoite invasion of erythrocytes is critical for blood-stage development; however, unlike in *P*. *falciparum*, blood-stage infection by *P*. *vivax* is limited to reticulocytes. An important mediator of this process is the Duffy binding protein (DBP), a parasite ligand released from micronenes of the apical complex just before reticulocyte invasion^8–10^. It is believed that DBP plays a dominant role during the irreversible step of junction formation just before invasion and interacts with its cognate receptor, the Duffy antigen receptor for chemokines (DARC) on the reticulocyte surface^11,12^. The vital nature of this interaction is evident in the very low prevalence of *P*. *vivax* in populations with high prevalence of Duffy negativity as in West Africa, thereby highlighting DBP as a promising target for vaccine-induced immunity. Although studies now confirm some *P*. *vivax* infections occur among DARC negative individuals^13–16^, this appears to be at low frequency. Currently, the mechanism used by the parasite to invade this group of individuals is still unknown but it is suggested that the parasite might have evolved to use Duffy-independent pathways for invading host erythrocytes that may use DBP paralog ligands^17,18^.

Asexual stage vaccine candidates especially those involved in erythrocyte invasion are often genetically diverse as a result of immune selection pressure, a mechanism used by the parasite to escape host immune response. This diversity in immune epitopes results in antigenically-distinct variants in the parasite population and the development of strain-specific immunity by limiting the induction of immune response towards more conserved protective epitopes. Such variation makes it difficult to design a single subunit vaccine that covers the full range of diversity, and could potentially facilitate the emergence of vaccine-resistant parasite strains. These strain-specific immune responses have been responsible for the failure observed in many *P*. *falciparum* vaccine candidates that have advanced to clinical trials, including MSP1^19^, PfMSP3^19,20^, PfAMA-1^21–23^, as well as other microbial pathogens such as the influenza hemagglutinin (HA)^24,25^ and the HIV ligand (gp120)^26,27^.

Multivalent vaccines are a successful strategy to overcome strain immunity to other microbial infections, using a combination of diverse alleles or strains to broaden the immune responses, although these can be a challenge to manufacture^28–30^. More recently, structure-based design using engineered immunogens has been pursued to focus immune protective responses on conserved epitopes^31,32^. An important obstacle in pursuing either of these types of vaccines, multivalent vaccines or structure-based design, is still our poor understanding of the basis of natural protective immunity thereby limiting our ability to select the best vaccine targets^33–35^. To overcome this important obstacle, *in vitro* functional assays that mimic the erythrocyte binding activity of the *P*. *vivax* Duffy binding protein together with immunochemical analyses and crystallography have been instrumental in identifying epitope targets of protective immunity and help guide a structure-based design^36–39^.

Region II of DBP (DBPII) is the critical adhesion ligand that participates in merozoite invasion of human Duffy-positive reticulocytes^36,40–42^. DBPII engages DARC in a stepwise fashion to create a stable heterotetramer of two DBP molecules and two DARC molecules^36,42^. Both the dimer interface of DBP and the DARC interaction site in DBP are targets of neutralizing antibody responses^36,38,42^. In addition, structural epitope mapping has identified epitopes in DBP for broadly-neutralizing and non-protective antibodies outside of the dimer interface and DARC binding residues^43^. DBPII is polymorphic, with substitution rate four times higher than the rest of the molecule^44–46^ in a pattern consistent with high immune selection pressure on this molecule^47^. Consequently, these polymorphisms represent a potential serious challenge that may compromise the efficacy of a DBP vaccine against diverse *P*. *vivax* strains. Previous studies have demonstrated that these naturally occurring polymorphisms mostly are not functionally important for receptor recognition but flank the functional residues important for receptor binding, suggesting that variation serves as a means of immune evasion^36,40–43,45^. Existing data also demonstrate that the naturally occurring polymorphisms in DBPII confer significant differences in sensitivity to inhibitory antibodies^48–50^. Similar to other microbial pathogens, anti-DBPII variant-specific antibody responses correlate with homologous but not heterologous protection^38,43,50–57^. Strain-transcending, broadly-neutralizing antibodies to DBP have been identified^38,43^. However, their low prevalence prompts the design of DBP-based immunogens that focus immune response to neutralizing epitopes. There is a significant correlation of their antibody reactivity to certain epitopes and inhibition of DBPII-receptor function. Nonetheless studies in endemic regions regularly find a few elite responders that have acquired strain-transcending anti-DBPII inhibitory responses^38,52,57,58^, indicating there are conserved immunogenic epitopes that are targets of broadly neutralizing anti-DBPII antibodies. These elite responders can be used as a tool to guide and validate the development of a strain-transcending DBPII vaccine.

In earlier proof-of-concept studies, we engineered a recombinant DEKnull as a novel DBPII immunogen to overcome the inherent bias towards developing strain-specific immunity^37,39,59,60^. DEKnull altered the charged/polar residues of the immunodominant variant B-cell epitope on DBPII to alanine, serine or threonine (DEKaQQRrKQ to AATaATSrTS)^37,39^. Recombinant DEKnull retained erythrocyte-binding activity and elicited functional antibodies to shared epitopes on the parent native Sal1 allele from which it was derived^39^. Although anti-DEKnull was shown to have lower inhibitory titres, it produced a more consistent strain-transcending inhibitory response than a native single or multi-allele DBPII immunogens^59^. This suggests that recombinant DEKnull contained conserved epitopes associated with natural protective immune response despite lacking the dominant epitope.

In the current study, functional antibodies elicited by the next generation engineered DBPII vaccine, DEKnull-2, were more immunogenic and induced more broadly-neutralizing antibodies against a range of diverse allelic DBPII variants than the original DEKnull. Importantly, DEKnull-2 has strong reactivity with long-term persistent antibody prevalent in acquired natural immunity to *P*. *vivax* infection of elite responders. These successful results indicate that focusing immune response to conserved functional epitope targets of strain-transcending immune responses is a potential approach that can be used generally to improve efficacy of other malaria vaccine candidates.

## Results

### Engineered DBPII immunogens

**Figure 1:**
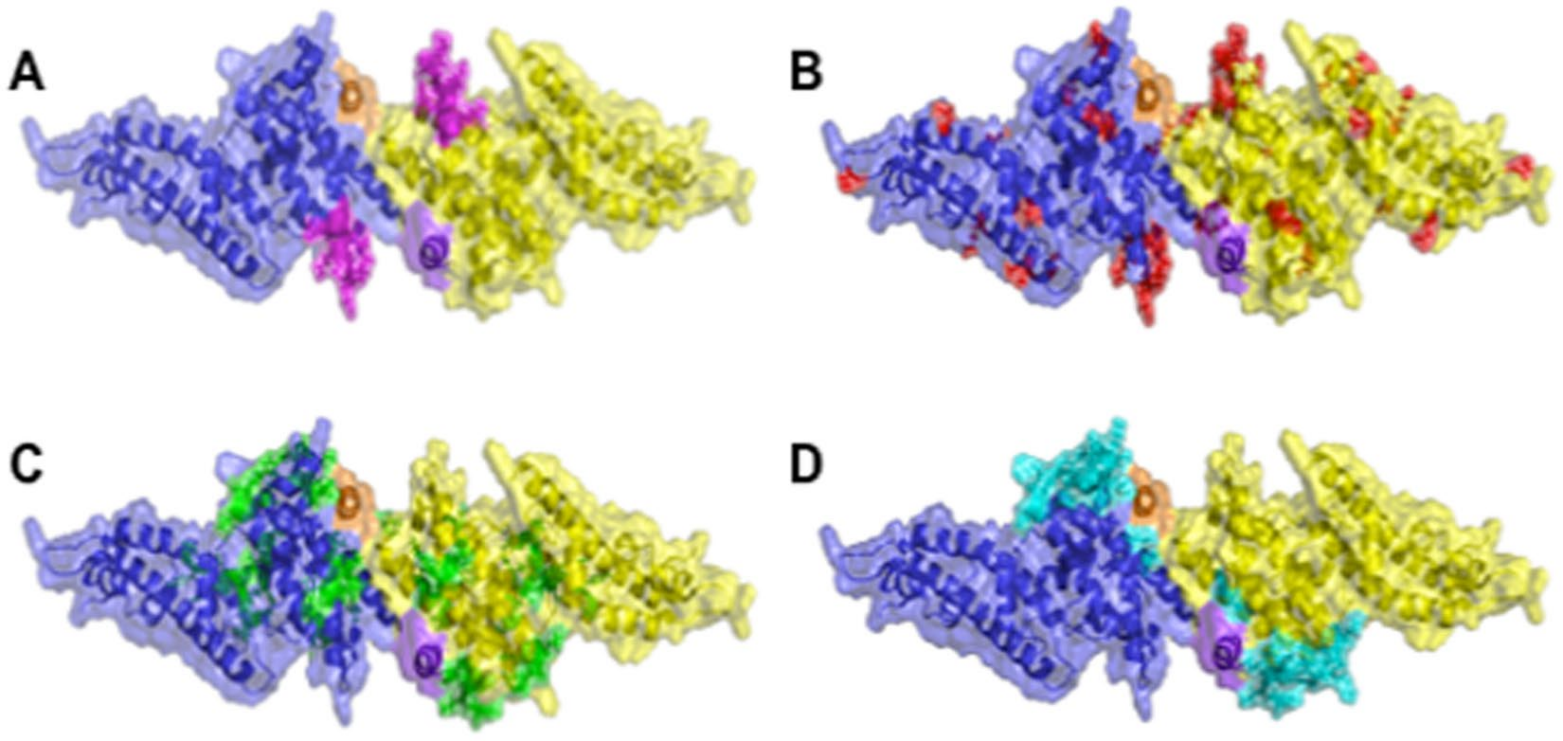
Homology model showing crystal structure of DBPII dimer. Monomers are represented in blue and yellow. Locations of mutated residues to create the various DEKnull antigens are indicated. (**A**) DEKnull: mutated immunodominant ‘DEK’ epitope, magenta (36); (**B**) DEKnull-2: mutated polymorphic residues, red; (**C**) DEKnull-3: mutated binding residues, green; and (**D**) DEKnull-4: mutated dimerization residues, cyan. The DARC binding groove is shown in orange and purple. The homology model structures were constructed using the PyMOL Molecular Graphics System software.

**Figure 2:**
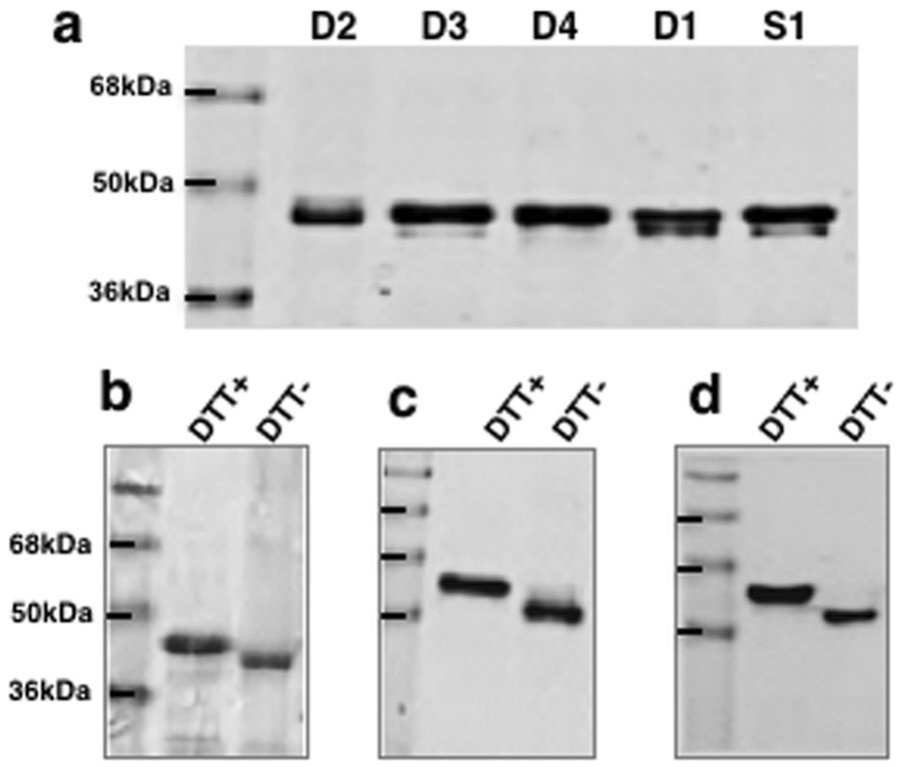
Production of recombinant antigens. (**a**) Scanned Coomassie-stained SDS-PAGE gel of recombinant DEKnull variants (DEKnull: D1; DEKnull-2: D2; DEKnull-3: D3; DEKnull-4: D4) and Sal1 (S1) purified by affinity chromatography on Ni + Sepharose resins. (**b**,**c**,**d**) Differential mobility of refolded recombinant DEKnull-2, DEKnull-3 and DEKnull-4 respectively on SDS-PAGE gel before (−) and after ( + ) reduction with DTT, is a simple indicator of presence of disulphide bonds in the refolded antigens. Production of recombinant Sal1 and DEKnull were previously reported^39^. Gels are cropped to improve clarity and conciseness of the figure. Full-length gels are presented in Supplementary Figure S1.

**Figure 3:**
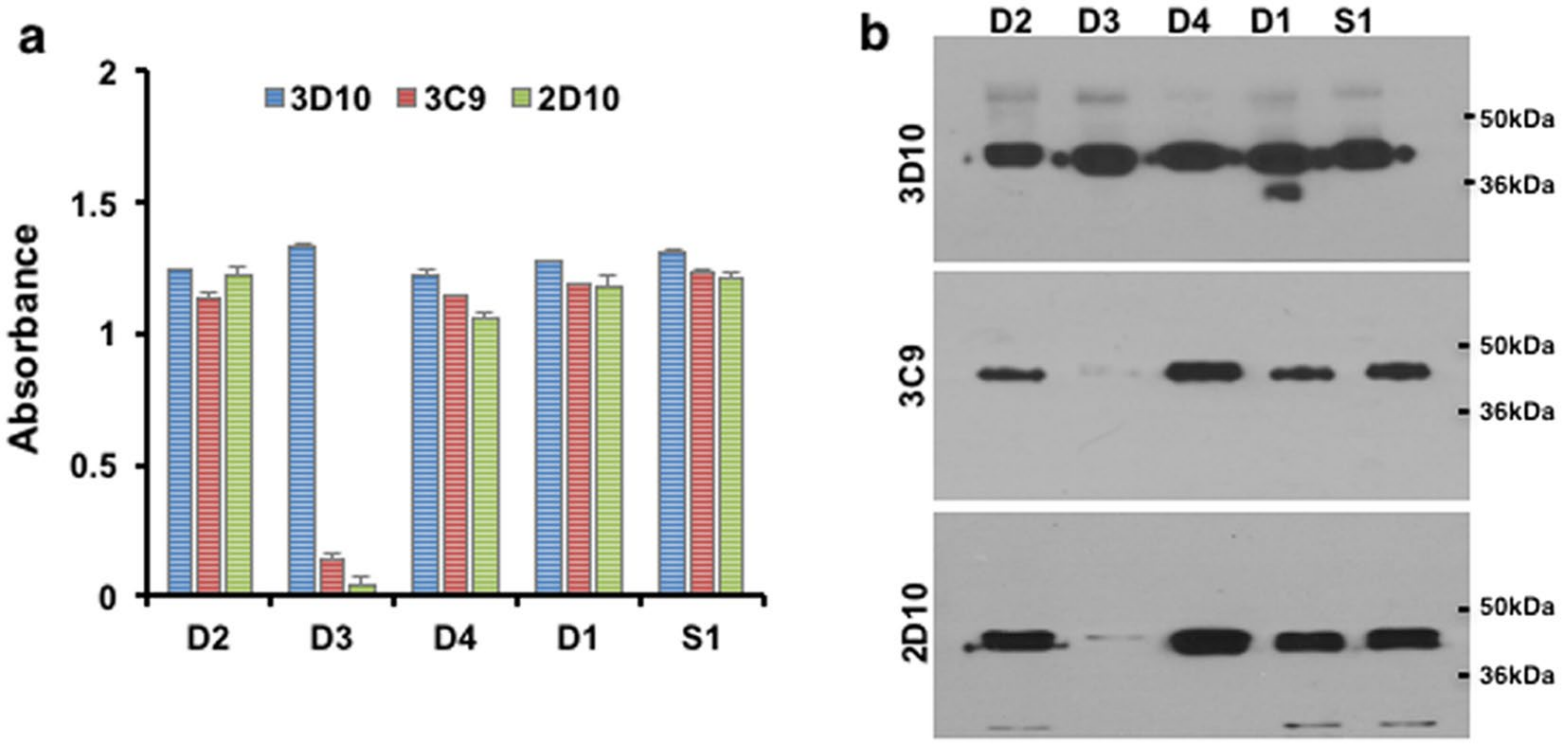
Reactivity of rDEKnull antigens with anti-DBPII monoclonal antibodies. Antigens were tested for reactivity with conformational dependent anti-DBPII mAbs 3C9 and 2D10 and non-conformational dependent mAb 3D10 by (**a**) ELISA and (**b**) Western blot analysis. Recombinant Sal1 was used as control antigen. Bars represent mean OD values. Error bars indicate ± standard deviation for triplicate wells. Blots are cropped to improve clarity and conciseness of the figure. Full-length blots are presented in Supplementary Figure S2.

**Figure 4:**
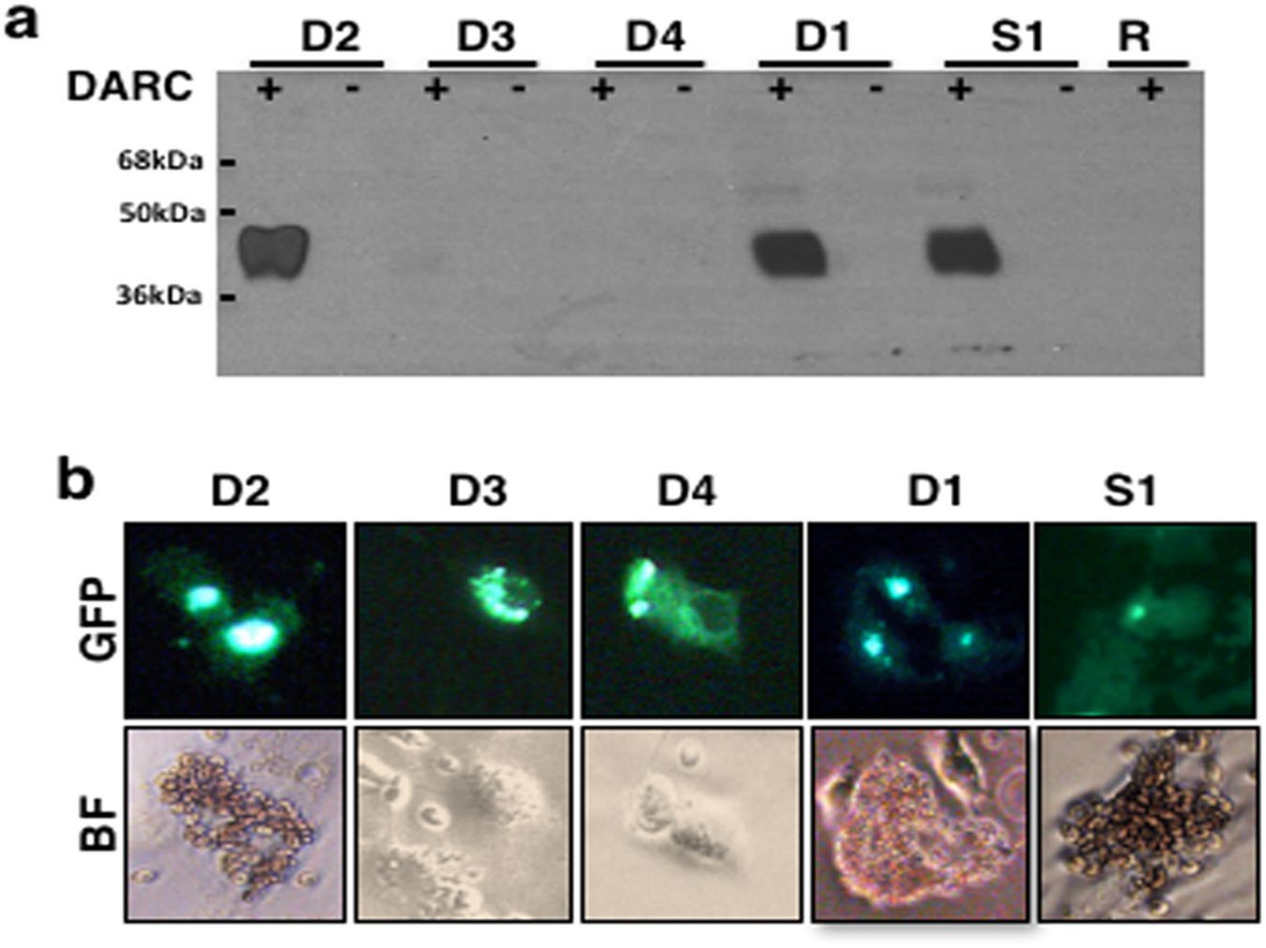
Functional analysis of the DEKnull antigens. (**a**) Western blot analysis shows refolded recombinant DEKnull (D1), DEKnull-2 (D2) and Sal1 (S1) bind to DARC positive ( + ) but not DARC negative (−) red blood cells, while recombinant DEKnull-3 (D3) and DEKnull-4 (D4) do not bind to either erythrocyte type. DARC positive red blood cells (R) without bound antigen served as control (**b**) COS7 cell surface-expressed Sal1 and DEKnull-2 antigens bind to DARC positive red blood cells in the standard *in vitro* COS7 cell binding assay, while DEKnull-3 and 4 do not. The blot is cropped to improve clarity and conciseness of the figure. The Full blot is presented in Supplementary Figure S3.

Three novel DBPII immunogens were engineered to alter the following types of residues: DEKnull-2, polymorphic^40^; DEKnull-3, determined by site-directed mutagenesis to be critical for erythrocyte binding^40,61^; and DEKnull-4, contact at dimer interface^36,42^ (Fig. 1 and Supplementary Table S1). Recombinant variant DEKnull proteins were expressed in *Escherichia coli*, purified from inclusion bodies, and refolded by rapid dilution (Fig. 2). Native conformation of the refolded proteins was confirmed using well-characterized conformation-dependent anti-DBPII monoclonal antibodies^48^, except rDEKnull-3 as expected (Fig. 3 and Supplementary Fig. S4). Functional evaluation with two standard *in vitro* assays, confirmed the correct erythrocyte binding activity of rDEKnull-2 and native rSal1 whereas as expected rDEKnull-3 and rDEKnull-4 did not bind erythrocytes (Fig. 4a and b).

### Immunogenicity

**Figure 5:**
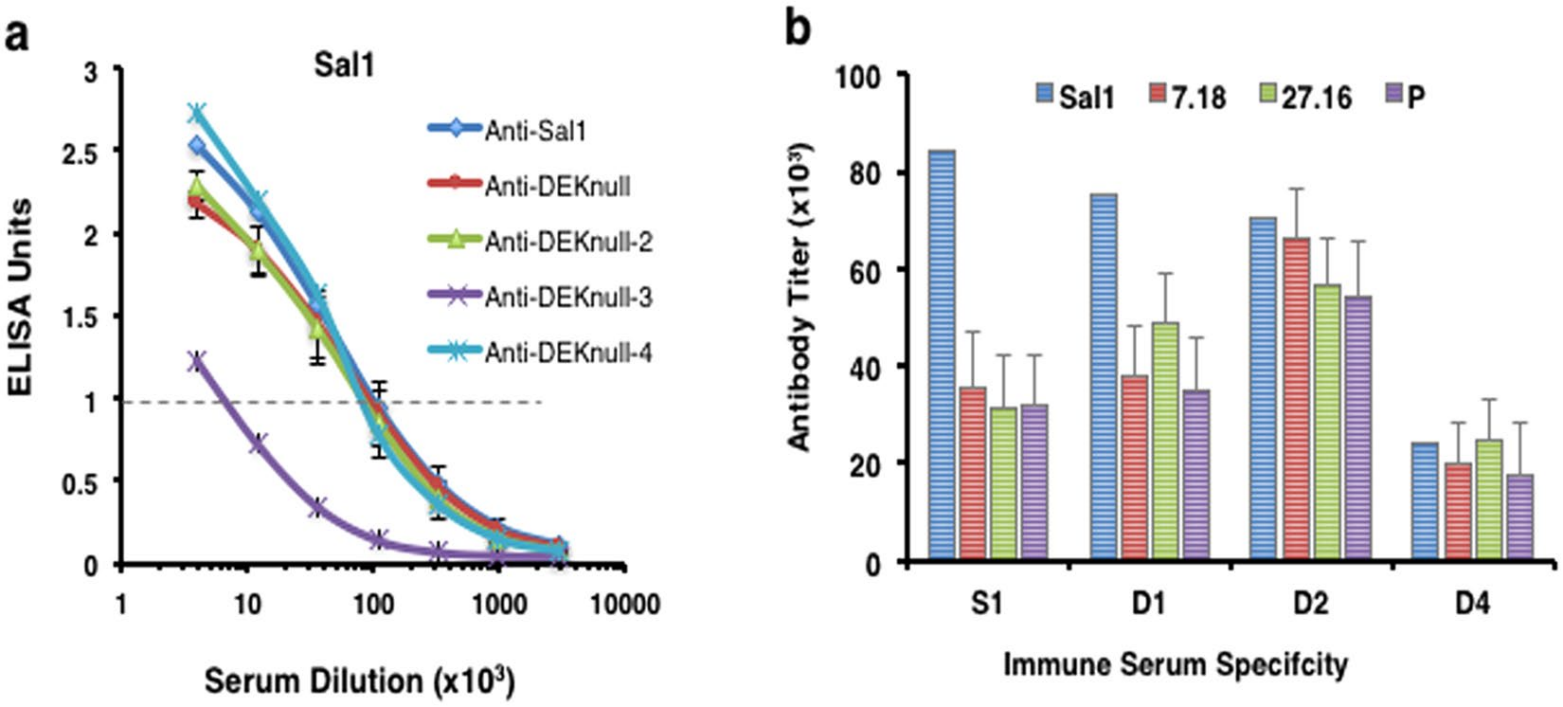
Anti-DBPII reactivity profiles. Reactivity of mouse antisera against the parent antigen Sal1 (**a**) and four naturally occurring variant DBPII alleles (Fig. S1) by end point titration ELISA. Each curve represents a 4-parameter logistic regression curve for antisera from each mouse (n = 15) against the different alleles (**b**) Quantitative comparison of reactivity profiles of the different antibodies to the variant DBPII alleles. Each bar represents the serum dilution at EU = 1.0. Error bars represent ± SD.

The immunogenicity of the engineered immunogens was evaluated by ELISA against the parent rSal1 antigen and three other naturally-occurring antigenically-distinct variant rDBPII alleles^59^. With the exception of rDEKnull-3, immune sera had highly reactive antibodies to the rDBPII alleles (Supplementary Fig. S5). Since DEKnull-3 was very poorly reactive to Sal1, its antiserum was not analyzed further for other variants. There was no significant difference in antibody response between anti-Sal1 and anti-DEKnull-2 sera against the parent rSal1 antigen (Fig. 5), but anti-DEKnull-2 reacted significantly better than anti-Sal1 to all four variant DBPII alleles (Fig. 5b). Anti-DEKnull-4 responses were significantly lower than that of anti-Sal1, anti-DEKnull and anti-DEKnull-2.

### Functional inhibition

**Figure 6:**
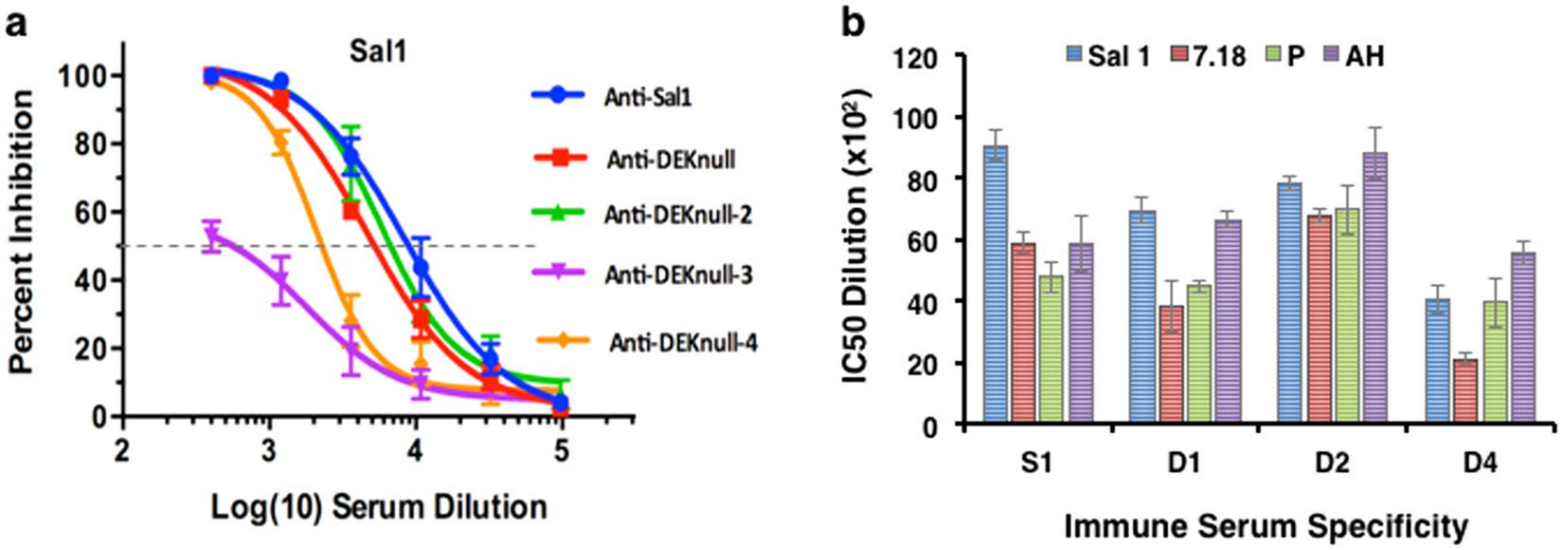
Inhibition of DBPII binding to DARC on human erythrocytes by standard COS7 cell assay. Antisera were tested for inhibition of DBPII-Sal1-erythrocyte binding (**a**) and three naturally occurring variant DBPII alleles (Spplementary Fig. S2) by end point dilution. Each curve on the chart represents non-linear regression of two independent experiments, with each dilution tested in triplicate and horizontal broken line shows the 50% inhibition (IC50). Graph was constructed using GraphPad Prism software. (**b**) Quantitative comparison of anti-DBPII inhibitory activity of the different sera against variant DBPII alleles based on the IC50 serum dilution. Error bars represent ± SD.

**Figure 7:**
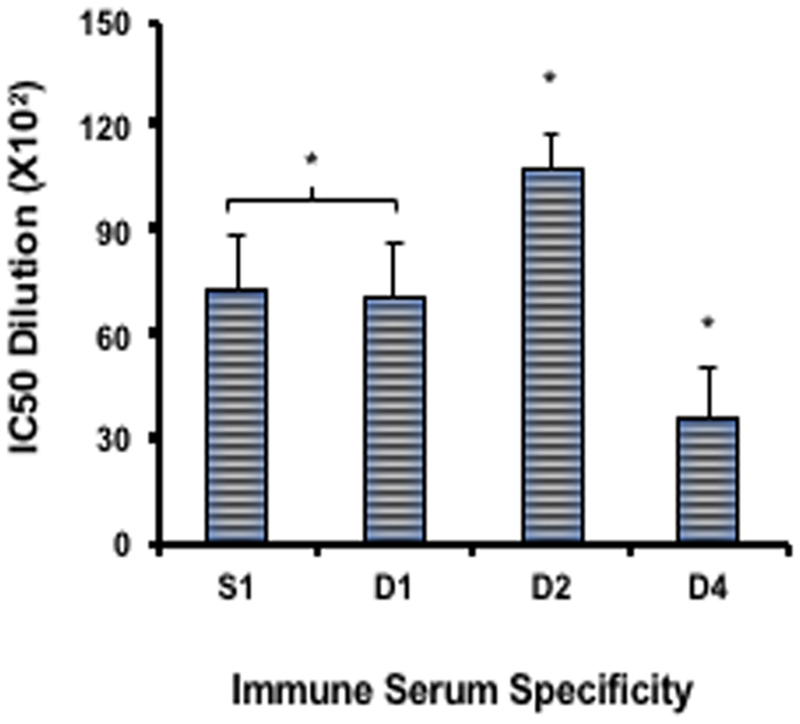
Multiple comparisons of anti-DBPII binding-inhibitory responses. The overall inhibitory response of each antiserum against all four COS7 cell-expressed DBPII alleles was compared with Dennett’s adjustment multiple comparisons, with Sal1 as control. Bars represent the mean IC50 value of each serum dilution against all the variant DBPII alleles tested in the COS7 assay. Sera were placed into three groups (DEKnull-2, Sal1/DEKnull, and DEKnull-4). Asterisk (*) indicates a significant difference in the inhibitory responses between the immune sera from the DEKnull-2 group and the DEKnull/Sal1 group and the DEKnull-4 group (*p* = 0.05).

The efficacy of each antiserum was tested for the presence of binding-inhibitory antibodies (BIAbs) by the ability to inhibit erythrocyte binding by DBPII-Sal1 and then three other naturally occurring DBPII variants (7.18, P, AH^59^). Anti-DBPII-Sal1, -DEKnull, and –DEKnull-2 sera had similar potent ‘homologous’ BIAb activity against the Sal1 allele (Fig. 6a), but anti-DEKnull-4 serum was significantly less inhibitory. Importantly, only anti-DEKnull-2 maintained a similar high-level of functional BIAbs against the antigenically-distinct DBPII variants of 7.18, P or AH (Fig. 6b and Supplementary Fig. S6). As expected anti-rDEKnull-3 was significantly lacking functional inhibition against Sal1 and therefore was not tested with the other variants. A Dunnett’s-adjusted multiple comparison was used to categorize the BIAb activity of the sera into three statistical groups, (DEKnull-2, Sal1 + DEKnull, DEKnull-4), using anti-rSal1 serum as control. Overall, DEKnull-2 vaccination elicited a significantly higher broadly-neutralizing BIAb response to variant DBPII than Sal1, DEKnull, or DEKnull-4 vaccines (*p* < 0.005) (Fig. 7).

### Naturally-acquired antibody responses to DEKnull-2

**Figure 8:**
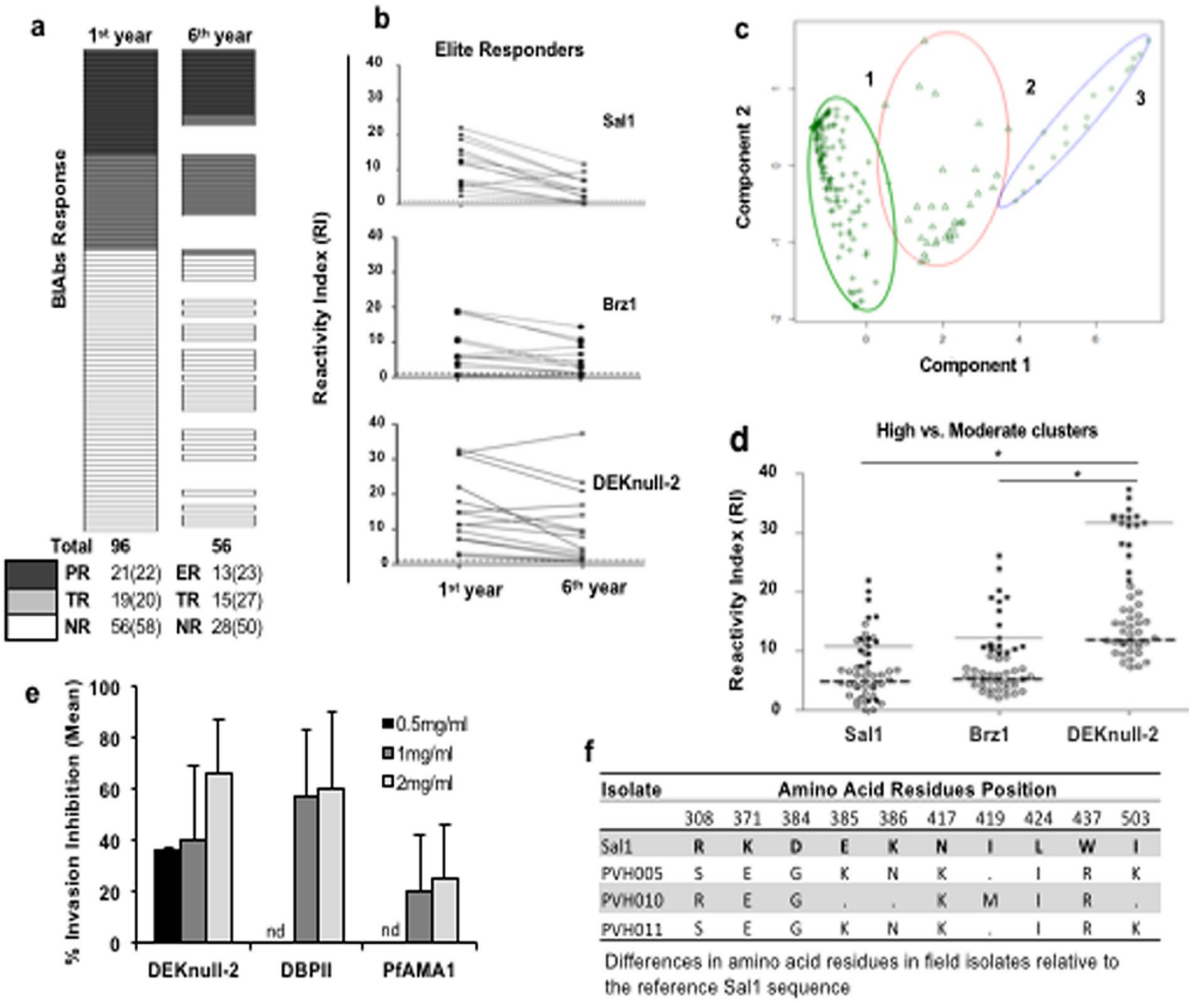
Naturally acquired immune response and anti-DBPII binding-inhibitory antibodies (BIAbs). (**a**) Plasma from study participants were screened for binding inhibitory antibodies (BIAbs) by COS7 cell assay. Non-responders (NR), had no BIAbs; Temporary responders (TP), had variable BIAbs; Persistent responders (PR), had BIAbs during all three cross-sectional surveys in the 1^st^ year, while Elite responders (ER), had BIAbs during all three cross-sectional surveys in the 1^st^ year and six years later. (**b**) IgG antibody profiles of individuals with long-term persistent antibody responders (ER) against naturally occurring DBPII Sal1, Brz1 and DEKnull-2. (**c**) K-means clustering analysis based on ELISA reactive index (RI) and BIAb activity of individual responses in each cohort during follow up from year one up till year six (n = 224) identifies three distinct clusters: 1. Low-responders, low RI and BIAbs (n = 173); 2. Moderate-responders, moderate RI and BIAbs (n = 34); and 3. High-responders, high RI and BIAbs (n = 17). (**d**) Total IgG specific antibodies to DBPII variants (Sal-1 and Braz1) and synthetic DEKnull-2 among the high (black cycle) and moderate-response groups (white cycle); Asterisk (*) indicates a significant difference between groups (p < 0.05; determined by Kruskal–Wallis test with Dunn’s post-test), with medians for high and moderate-response clusters represented by continuous or discontinuous lines. (**e**) Inhibition of *P*. *vivax* invasion of human reticulocytes by anti-DEKnull-2 and anti-DBPII-Sal1. Invasion was determined by analyzing newly invaded rings in wells of culture plates in the presence of antibodies relative to wells with no antibodies. The bars represent mean percentage invasion-inhibition against all three different isolates and error bars represent ± standard deviation. (nd: not done). (**f**) Differences in amino acid residues in DBPII between the three field isolates relative to the Sal1 reference sequence.

To investigate whether epitopes of the engineered DEKnull-2 vaccine were antigenic in natural immunity to *P*. *vivax* malaria, we measured total IgG antibodies to recombinant DEKnull-2 and two naturally occurring DBPII alleles (Sal1, and a common Brazilian variant, Brz1). These sera from residents living in a well-characterized rural population in a malaria-endemic area of the Brazilian Amazon were characterized for functional BIAbs to Brz1 (Fig. 8). Individuals (n = 96) with: no BIAb activity were classified as non-responders (NR); with variable BIAb activity as temporary responders (TR); and with BIAb activity during all three cross-sectional surveys in the 1^st^ year as persistent responders (PR). A subset of PR individuals is referred to as “elite responders” (ER) because they were determined to maintain detectable levels of anti-DBPII BIAbs in all three first-year cross-sectional studies and six years later (Fig. 8a). Interestingly, the anti-DEKnull-2 IgG titres in the ER individuals was much higher and more stable than those against the naturally-occurring strain-specific antibody response to Sal1 and Brz1 DBPII variants (Fig. 8b). To confirm the immune response to DEKnull-2, we performed a K-means clustering analysis, which allowed classification of each sample (n = 224), (fifty-six individuals followed through all four cross-sectional studies), based both on IgG antibody responses (RI) and DBPII-specific BIAbs. Three distinct immune response clusters were identified and classified as: low responders, low RI and BIAbs (n = 173); moderate responders, moderate RI and BIAbs (n = 34); and high responders, high RI and BIAbs (n = 17) responders (Fig. 8c). This clustering analysis further confirms that these immune individuals had significantly higher naturally-acquired antibody responses to the engineered DEKnull-2 vaccine than to Sal1 and Brz1 (*p < 0*.05) (Fig. 8d).

### Inhibition of merozoite-reticulocyte invasion

Immune serum IgG to rDEKnull-2 and rSal1 were evaluated for ability to inhibit merozoite invasion of human reticulocytes in a short-term *in vitro P*. *vivax* cultures (Fig. 8e). There was a dose-dependent inhibition of parasite invasion of reticulocytes by the anti-DEKnull-2 antibodies against parasite isolates from three different patients (Fig. 8f), ranging from 36% inhibition at 0.5 mg/ml to 66% inhibition at 2 mg/ml, while the anti-Sal1 antibody inhibited invasion by 57% and 60% at 1 and 2 mg/ml, respectively. A *P*. *falciparum* antibody (anti-PfAMA1) used as antibody control showed 20 and 23% inhibition of invasion at 1 and 2 mg/ml respectively while anti-Fy6, a DARC specific monoclonal antibody inhibited invasion by ≈83% to 85% at 25 µg/ml and 50 µg/ml respectively (not shown).

## Discussion

*Plasmodium vivax* merozoite invasion of reticulocytes is a rapid complex process involving interactions between the DBP and its cognate receptor DARC^11^. Since this interaction is vital for maintaining vivax malaria in a population, DBP represents an attractive target for vaccine development. However, naturally-acquired anti-DBPII antibodies tend to be poorly inhibitory, short-lived, unstable, and biased towards strain-specific responses^38,57^. Even so, the less prevalent strain-transcending, broadly neutralizing antibodies can confer protective anti-DBP immunity by targeting epitopes at or near the dimer interface of DBPII, thereby blocking DBP dimerization and inhibiting invasion^36,38,42,43^. The weakness and strain-specificity of natural immunity elicited by *P*. *vivax* infection is presumed to occur because these less immunogenic conserved functional epitopes at the dimer interface are flanked by more immunogenic but variable residues^36–38,42,43^.

The goal of this project is to overcome the natural tendency of DBPII to elicit strain-specific responses. Therefore, recombinant DBPII immunogens were engineered to ablate variant strain-specific epitopes to broaden efficacy by immunofocusing acquired responses on conserved epitopes that could be targets of strain-transcending BIAb inhibition of diverse DBPII alleles. Building on proof-of-concept studies that engineered alteration of a single dominant variant B-cell epitope of DBPII^37,39,59^, the current study extensively engineered DEKnull-2 with substitutions for most of the predicted polymorphic surface residues not important for erythrocyte binding function^40^. Importantly, the newly engineered rDEKnull-2 maintains its native conformation and DBP functional activity by binding to DARC on human erythrocytes.

Vaccination with DEKnull-2 induced a significantly higher titre response to all the variant DBPII alleles compared to the Sal1 and the original DEKnull. Similarly, overall anti-DEKnull-2 BIAb responses to variant DBPII alleles were significantly higher than BIAb elicited by vaccination with native Sal1 and the DEKnull (*p < 0*.05) (Fig. 7). These data indicate that the DEKnull-2 vaccine, which lacks the polymorphic residues in DBPII, focused the immune response to the conserved functional epitopes on DBPII. Additional analyses, using other engineered DBPII immunogens, confirmed that overall conformation of the DBL ligand domain and especially retention of the functional contact residues needed for DBPII dimerization on receptor binding are critical for induction of broadly-neutralizing BIAb. Altogether these data confirm the hypothesis that conserved functional residues in DBPII important for erythrocyte binding are the targets of protective strain-transcending immunity.

Humoral immune responses to blood-stage antigens are believed to be important for naturally-acquired immunity to malaria and to be most effective vaccines need to replicate these protective mechanisms^62,63^. Therefore, we evaluated sera from residents living in a malaria-endemic agricultural settlement of the Brazilian Amazon for the presence of naturally-acquired IgG antibodies to the DEKnull-2 vaccine. The highest level of naturally-acquired immune antibody responses detected against DEKnull-2 were during the first year and these antibody responses remained stable during six years of follow up, even in the absence of clinical disease or infection (Fig. 8). To evaluate the correlation between anti-DBP antibody response and functional inhibition of DBPII-erythrocyte binding, individual serum was tested for presence of BIAb. The elite responders with long-term high-titre anti-DEKnull-2 antibodies (reactivity index > 20 in ELISA) had stable BIAb throughout the study period. Correspondingly, potential efficacy of a DEKnull-2 vaccine was further demonstrated by inhibition of merozoite invasion of reticulocytes by anti-DEKnull-2 antibodies in short-term *ex vivo* parasite cultures. These data indicate that conserved functional epitopes of DEKnull-2 are naturally immunogenic, supporting its use as a potential protective vaccine.

In summary, we engineered a novel DBPII vaccine DEKnull-2 by extensively altering the polymorphic residues on the DBP ligand domain. We demonstrate that this immunogen elicits a higher titre and more broadly neutralizing anti-DBPII BIAb against variant DBPII alleles than the current vaccine candidate DBPII-Sal1 and these BIAb block *P*. *vivax* merozoite invasion of human reticulocytes. Most importantly, conserved functional epitopes on DEKnull-2 are the target of naturally-acquired BIAbs that persist long-term protective immune responses in *P*. *vivax*–exposed individuals. Our data confirm that epitope specificity is critical in vaccine design and induction of a strain-specific responses can be eliminated. Finally, antigen engineering to immunofocus responses to conserved functional regions, such as the DBPII dimer interface, is a viable and practical approach. These results indicate a potential approach that can be used generally to improve efficacy of other malaria vaccine candidates.

## Materials and Methods

### Immunogen design and recombinant antigen production

The production of recombinant DBPII-Sal1, DEKnull, and other variant DBPII alleles was previously reported^39,59^. The Sal1 sequence was used as a template to create three novel DBPII antigens by substituting (i) polymorphic residues^40^ (ii) binding residues^40^ and (iii) residues important for dimerization^36^ with non-polar or non-charged residues such as alanine, threonine or serine to produce the antigens DEKnull-2, DEKnull-3 and DEKnull-4 respectively (Fig. 1 and Supplementary Table S1). The sequences coding for these three novel antigens were codon-optimized for *E*. *coli* expression and the DNA commercially synthesized and cloned into pET21a + expression vector (Novagen), with a C-terminal 6xHis tag to facilitate purification by affinity chromatography. Protein expression was carried out in *E*. *coli* BL21(DE3) LysE cells (Invitrogen) after induction with 1 mM IPTG final concentration for 3 hr at 30 °C. Expressed proteins were purified from inclusion bodies by affinity chromatography using Ni + Sepharose 6 fast flow (GE Lifesciences) and finally refolded by rapid dilution as previously reported^39,59,64^.

### Functional analysis of refolded antigens

A standard *in vitro* erythrocyte-binding assay was used to test for functional ligand activity^59,64^. Briefly, recombinant antigens were incubated with Duffy-positive and Duffy-negative erythrocytes. Bound antigens were eluted from the erythrocyte surface with 300 mM NaCl. Eluted proteins were separated by SDS-PAGE, transferred on to nitrocellulose membrane and probed with an anti-DBPII antibody, mAb-3D10^48^.

### Immunizations

Polyclonal antibodies were raised in 6–8 weeks old BALB/c mice obtained from Envigo, USA and housed in specific pathogen-free conditions. All animals were handled in compliance with good animal practice and all experimental procedures were performed in accordance with protocols, guidelines and regulations approved by the Institutional Animal Care Use Committee (IACUC), Division of Research Integrity and Compliance, University of South Florida. Groups of mice (n = 15) were each immunized twice at three-week intervals either with 25 µg of recombinant Sal1, DEKnull, DEKnull-2, DEKnull-3 or DEKnull-4 emulsified in Titermax Gold adjuvant (Titermax) as previously reported^59^. A 50 µl antigen-adjuvant mixture was injected subcutaneously at the base of the tail and serum samples were collected three weeks after second immunization by exsanguination and stored at −20 °C until needed. Mice immunized with PBS mixed with adjuvant alone, as well as pre-immune sera, served as controls.

### Measurement of antibody titres

Total anti-DBPII IgG titres in the serum of each mouse was evaluated by end-point titration ELISA against naturally occurring variant rDBPII alleles Sal1, 7.18, P and 27.16^48^ and the corresponding homologous antigens. A DBPII specific mAb-3D10^48^ was used as standard on each plate. Briefly, wells of micro titre plates were coated with 0.2 µg of antigen overnight and blocked with 5% skimmed milk before incubating with 3-fold dilution of mouse sera as previously reported^59^. Bound antibodies were then detected with an alkaline phosphatase-conjugated anti-mouse antibody (KPL). All OD values were expressed as ELISA units (EU), determined as a ratio of the OD_650_ generated by the test antibody and OD_650_ ≅ 1.0 of the standard^59^. Pre-immune sera and sera from mouse immunized with adjuvant alone served as negative controls.

### Measurement of functional inhibition of DBP-erythrocyte binding

To evaluate inhibitory activity of the mouse immune sera to DBPII-erythrocyte binding, a panel of naturally occurring DBPII alleles: Sal1, 7.18, 27.16 and P^59^ were transiently expressed on the surface of transfected COS7 cells as previously described^48,65^ and transfected cells were pre-incubated with triple-fold dilution of pooled mouse serum from each group prior incubating with human Duffy-positive erythrocytes. Binding was quantified by counting rosettes observed in 30 microscope fields (x200). Percent binding-inhibition was quantified by assessing the percentage of rosettes in wells of transfected cells in the presence of test serum relative to rosettes in wells in the presence of pre-immune serum.

### Measurement of naturally acquired anti-DBP antibody responses and anti-DBPII erythrocyte-binding-inhibitory antibodies

Plasma samples for this study were obtained from malaria-exposed inhabitants of an agricultural settlement of Rio Pardo, in Presidente Figueiredo municipality, Amazonas State, Brazil. The study was approved by the Ethical Committee of Research on Human Beings from the Centro de Pesquisas Renè Rachou (Report No. 007/2006 and No. 07/2009), according to the Resolution of the Brazilian Council on Health-CNS 196 / 96 after consultation with the community. Written informed consent was obtained from adults, caregivers and guardians of participating minors. Plasma IgG antibody titres to recombinant DBPII Salvador1 (Sal1), Brazil1 (Brz1) and DEKnull-2 was quantified by standard enzyme-linked immunosorbent assay (ELISA) as previously described^66^. ELISA data were expressed as reactivity index (RI), calculated by dividing the OD values of test sera by background reactivity (mean OD values of 30 naïve individuals plus 3 STD). To evaluate the potential binding-inhibitory activity of the samples, each plasma samples was tested for inhibition of DBPII-erythrocyte binding at 1:40 dilution by the standard COS7 cell assay as described above. A pool of *P*. *vivax* immune serum and naïve serum was used as positive and negative control sera respectively. The percent binding-inhibition was calculated as described above. Plasma samples with more than 50% inhibition of DBPII-erythrocyte binding were considered inhibitory. Study population and detailed methods are provided in Supplementary material S1.

### Invasion-inhibition assay

Short term *in vitro P*. *vivax* cultures were performed with parasites isolated from patients attending the Tropical Medicine Centre Rondônia (CEMETROM) in Porto Velho or the outpatient Malaria Centre at Candeias do Jamari, Rondônia, Brazil. Blood was collected by venipuncture from patients with positive Giemsa-stained blood smears into citrate tubes after informed consent from adult patients or guardians of participating minors, in accordance with methods, guidelines and regulations approved by the Ethical Committee of the Centro de Pesquisas René Rachou (CAAE: 50522115.7.0000.5091). Invasion assays were performed as previously reported^67^. Briefly, purified schizonts infected red blood cells (iRBCs) were re-suspended at 1:1 ratio with reticulocytes and adjusted to 4% hematocrit with McCoy5A complete media. iRBCs-reticulocyte mix was added to each well of 96-well plates in the presence or absence of test antibodies (total IgG) at three different concentrations in triplicate. The plates were incubated at 37 °C in the presence of 90% N_2_, 5% CO_2_, 5% O_2_ mixed gas for 12 hr. Invasion-inhibition was quantified as percentage of ring-iRBCs in wells with test antibodies relative to wells with IgG from pre-immune serum. Detailed methods are provided in Supplementary material S1.

### Statistical analysis

A 4-parameter logistic regression was used to provide dose-response curves and the end-point titre for each antibody. Based on the dose-response curves, an ELISA unit of 1.0 (on the log-phase of the curve) was chosen for all further comparisons of the antibody titre. Since the data were not normally distributed, a Kruskal Wallis was used to determine if there are overall significant differences between the serum antibodies, followed by a Bonferroni multiple comparison (SAS). A non-linear regression was used to provide the dose-response curves for the anti-DBPII inhibitory concentrations (GraphPad). IC50s were calculated based on the percent inhibition and were compared for overall significant differences using the Kruskal-Wallis. As a follow-up, a Dunnett’s adjusted multiple comparison was used, with Sal1 as the control for the multiple comparison (SAS).

For the naturally acquired antibodies, the differences in medians of the RI ELISA were tested using either the Mann-Whitney or Kruskal–Wallis tests, with Dunn’s post hoc test, as appropriate. K-means clustering analysis was used to group the response of samples where the variables are the ELISA reactive index, for each antigen (Sal1, Brz1 and DEKnull-2) and binding-inhibition antibody activity.

## Electronic supplementary material

Supplementary Information
