# Supplementary material for: An engineered vaccine of the Plasmodium vivax Duffy binding protein enhances induction of broadly neutralizing antibodies
Source: Sci Rep. 2017 Oct 23;7:13779. doi: 10.1038/s41598-017-13891-2 (PMC5653783; doi:10.1038/s41598-017-13891-2)
Supplement: Supplementary file 1 — Supplementary Information [file 41598_2017_13891_MOESM1_ESM.pdf]

An engineered vaccine of the *Plasmodium vivax* Duffy binding protein enhances induction of broadly neutralizing antibodies

Francis B. Ntumngia[1], Camilla V. Pires[2], Samantha J. Barnes[1], Miriam T. George[1], Richard Thomson-Luque[1], Flora S. Kano[2], Jessica R. S. Alves[2], Darya Urusova[3], Dhelio B. Pereira[4], Niraj H. Tolia[3], Christopher L. King[5], Luzia H. Carvalho[2], John H. Adams[1]*.

[1]Center for Global Health and Infectious Diseases Research, Department of Global Health, College of Public Health, University of South Florida, Tampa, 33612, USA

[2]Centro de Pesquisas René Rachou/FIOCRUZ, Belo Horizonte, 30190, Brazil

[3]Departments of Molecular Microbiology & Microbial Pathogenesis, and Biochemistry & Molecular Biophysics, Washington University School of Medicine, Saint Louis, 63130, USA

[4]Centro de Pesquisa em Medicina Tropical de Rondonia-CEPEM, Porto Velho, 76812-245, Brazil[d]

[5]Center for Global Health and Diseases, Case Western Reserve University, Cleveland, 44106, USA

*Corresponding author: J. H. Adams. 3720 Spectrum Blvd., Suite 404, Tampa, FL 33612. Tel: (813) 974-9916. Fax: (813) 974-0992

E-mail: usfmalaria@gmail.com

Supplementary Methods Text S1

Study population and naturally acquired anti-DBP antibody responses and DBPII-erythrocyte binding-inhibitory antibodies. Human plasma samples for this study were obtained from malaria-exposed inhabitants of an agricultural settlement of Rio Pardo, in Presidente Figueiredo municipality, Amazonas State, Brazil. The study site and malaria transmission patterns have been described elsewhere [1-3]. Inhabitants of the area live on subsistence farming and fishing along small streams. Although both *P. vivax* and *P. falciparum* are transmitted throughout the year, *P. vivax* is responsible for about 90% of malaria cases. The study was approved by the Ethical Committee of Research on Human Beings from the Centro de Pesquisas Renè Rachou (Report No. 007/2006 and No. 07/2009), according to the Resolution of the Brazilian Council on Health-CNS 196 / 96. Written informed consent was obtained from adults or next of kin, caregivers, and guardians of participating minors. A population-based open cohort study was initiated in November 2008, and six and twelve months after the initial survey. Six years later, one more cross-sectional survey was conducted, prioritizing areas/groups with well-defined anti-DBPII immune responses. All surveys follow well-established protocols, including epidemiological and clinical interviews, previously described by [1-3]. A total of 96 individuals were enrolled in the first year and 56 during the sixth year follow up evaluations. Blood samples were collected by venipuncture from each volunteer in to EDTA tubes. Plasma IgG antibody titers to recombinant DBPII Salvador1 (Sal1), Brazil1 (Brz1) and DEKnull-2 was quantified by standard enzyme-linked immunosorbent assay (ELISA) as previously described [4]. Briefly, micro titer plates were coated with 300 ng of antigen per well and plasma samples tested at 1:100 dilutions. ELISA data were expressed as reactivity index

(RI), calculated by dividing the OD values of test sera by background reactivity (mean OD values of 30 naïve individuals plus 3 SD). Samples with RI > 1.0 were considered positive. To evaluate the potential binding-inhibitory activity of the samples, each plasma samples was tested for inhibition of DBPII-erythrocyte binding at 1:40 dilution by the standard COS7cell assay as described above. Binding was quantified by counting rosettes observed in 10–20 fields of view (x200). A pool of plasma from individuals with long-term exposure to malaria in the Amazon area were used as positive control, while pooled plasma samples from Rio Pardo residents (n=10) characterized as non-responders against DBPII by ELISA were used as negative control. The percent inhibition was calculated as 100 x (Rc—Rt)/Rc, where Rc is the average number of rosettes in the negative control wells, and Rt is the average number of rosettes in the test wells. Plasma samples with more than 50% inhibition of DBPII-erythrocyte binding were considered inhibitory.

Invasion-inhibition assay. Short term *in vitro P. vivax* cultures were performed with parasites isolated from patients attending the Tropical Medicine Centre Rondônia (CEMETROM) in Porto Velho or the outpatient Malaria Centre at Candeias do Jamari, Rondônia, Brazil. Blood was collected by venipuncture from patients with positive Giemsa-stained blood smears into citrate tubes after informed consent using protocols approved by the Ethical Committee of the Centro de Pesquisas René Rachou (CAAE: 50522115.7.0000.5091). Leukocytes were depleted from infected blood by filtration using cellulose columns (Sigma) [5]. Schizont-stage parasites were isolated by 45% Percoll (Sigma) gradient [6] and invasion assays performed as previously reported [7]. Briefly, reticulocytes were isolated from peripheral blood (Interstate blood bank) by centrifugation with 19% Nycodenz [8]. Schizonts infected red blood cells (iRBCs) were re-suspended at

1:1 ratio with reticulocytes and adjusted to 4% hematocrit with McCoy5A complete media. 200 µL of the iRBCs-reticulocyte mix was added to each well of 96-well plates. The plates were incubated at 37°C in the presence of 90% N_2, 5% CO_2, 5% O_2 mixed gas. For invasion-inhibition assays, parasites were incubated in the presence or absence of anti-DEKnull-2 and anti-DBPII-Sal1 antibodies (total IgG) at three different concentrations in triplicate wells. Invasion events (exclusively newly formed rings) were counted in 5 x 10^3 cells by microscopic examination of Giemsa-stained thin smears 12 hr post incubation.

Immunofluorescence assay. Transfected COS 7 cells were incubated on cover slips in a 24 well plate for 42 hr [9]. The cells were then fixed with PBS/2% formaldehyde for 15 min, washed 3x with PBS and incubated at room temperature with 5 µg/ml of anti-DBPII monoclonal antibody, mAb-3C9 for 90 min. After three washes with PBS, the cells were incubated with rhodamine-conjugated goat anti-mouse secondary antibody in PBS/0.1%BSA for 30 min in a dark moist chamber. Cell DNA was counter stained with DAPI. After three washes, the cells were observed by fluorescence microscopy.

References

1 Kano, F. S. *et al.* Plasmodium vivax Duffy binding protein: baseline antibody responses and parasite polymorphisms in a well-consolidated settlement of the Amazon Region. *Trop Med Int Health* **17**, 989-1000, doi:10.1111/j.1365-3156.2012.03016.x (2012).

2 Kano, F. S. *et al.* The Presence, Persistence and Functional Properties of Plasmodium vivax Duffy Binding Protein II Antibodies Are Influenced by HLA Class

II Allelic Variants. *PLoS neglected tropical diseases* **10**, e0005177, doi:10.1371/journal.pntd.0005177 (2016).

3 Souza-Silva, F. A. *et al.* Duffy antigen receptor for chemokine (DARC) polymorphisms and its involvement in acquisition of inhibitory anti-duffy binding protein II (DBPII) immunity. *PLoS One* **9**, e93782, doi:10.1371/journal.pone.0093782 (2014).

4 Ceravolo, I. P. *et al.* Anti-Plasmodium vivax duffy binding protein antibodies measure exposure to malaria in the Brazilian Amazon. *The American journal of tropical medicine and hygiene* **72**, 675-681 (2005).

5 Sriprawat, K. *et al.* Effective and cheap removal of leukocytes and platelets from Plasmodium vivax infected blood. *Malaria journal* **8**, 115, doi:10.1186/1475-2875-8-115 (2009).

6 Lim, C. *et al.* Expansion of host cellular niche can drive adaptation of a zoonotic malaria parasite to humans. *Nat Commun* **4**, 1638, doi:10.1038/ncomms2612 (2013).

7 Russell, B. *et al.* A reliable ex vivo invasion assay of human reticulocytes by Plasmodium vivax. *Blood* **118**, e74-81, doi:10.1182/blood-2011-04-348748 (2011).

8 Roobsoong, W. *et al.* Improvement of culture conditions for long-term in vitro culture of Plasmodium vivax. *Malaria journal* **14**, 297, doi:10.1186/s12936-015-0815-z (2015).

9 Chitnis, C. E. & Miller, L. H. Identification of the erythrocyte binding domains of Plasmodium vivax and Plasmodium knowlesi proteins involved in erythrocyte invasion. *J Exp Med* **180**, 497-506 (1994)

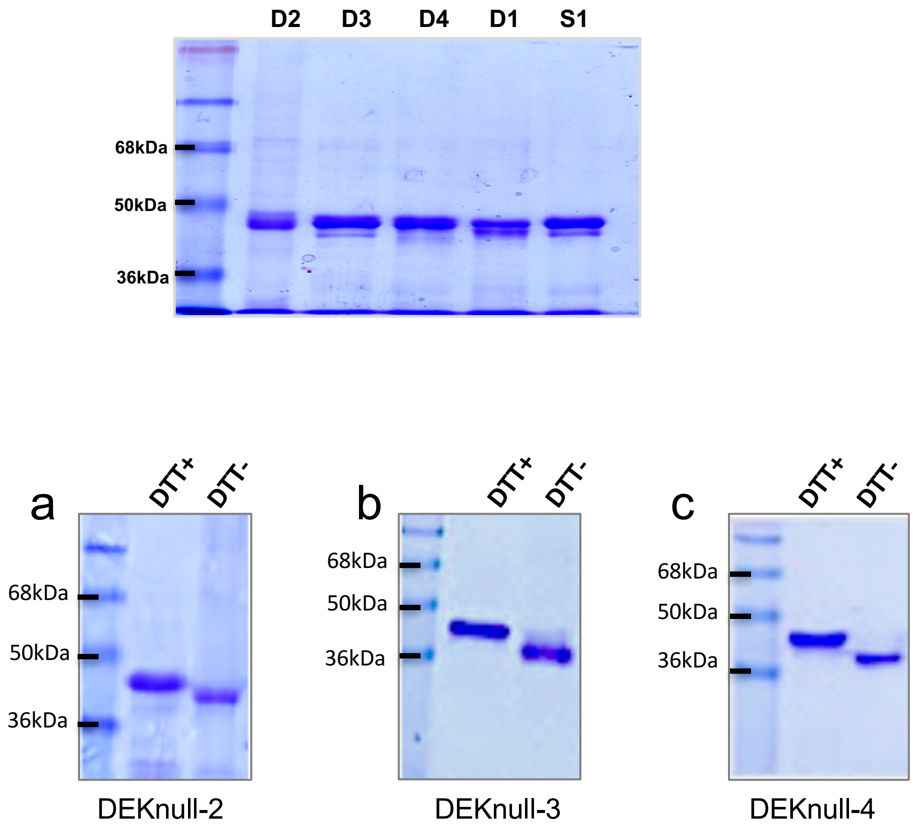

Supplementary Figure S1. Production of recombinant antigens. (**a**) Original Coomassie-stained SDS-PAGE gel of recombinant DEKnull variants (DEKnull: D1; DEKnull-2: D2; DEKnull-3: D3; DEKnull-4: D4;) and Sal1 (S1) purified by affinity chromatography on Ni+ Sepharose resins. (**b, c, d**) Differential mobility of refolded recombinant DEKnull-2, DEKnull-3 and DEKnull-4 respectively on SDS-PAGE gel before (-) and after (+) reduction with DTT, is a simple indicator of presence of disulphide bonds in the refolded antigens. Production of recombinant Sal1 and DEKnull were previously reported [40]. Images represent scanned gels.

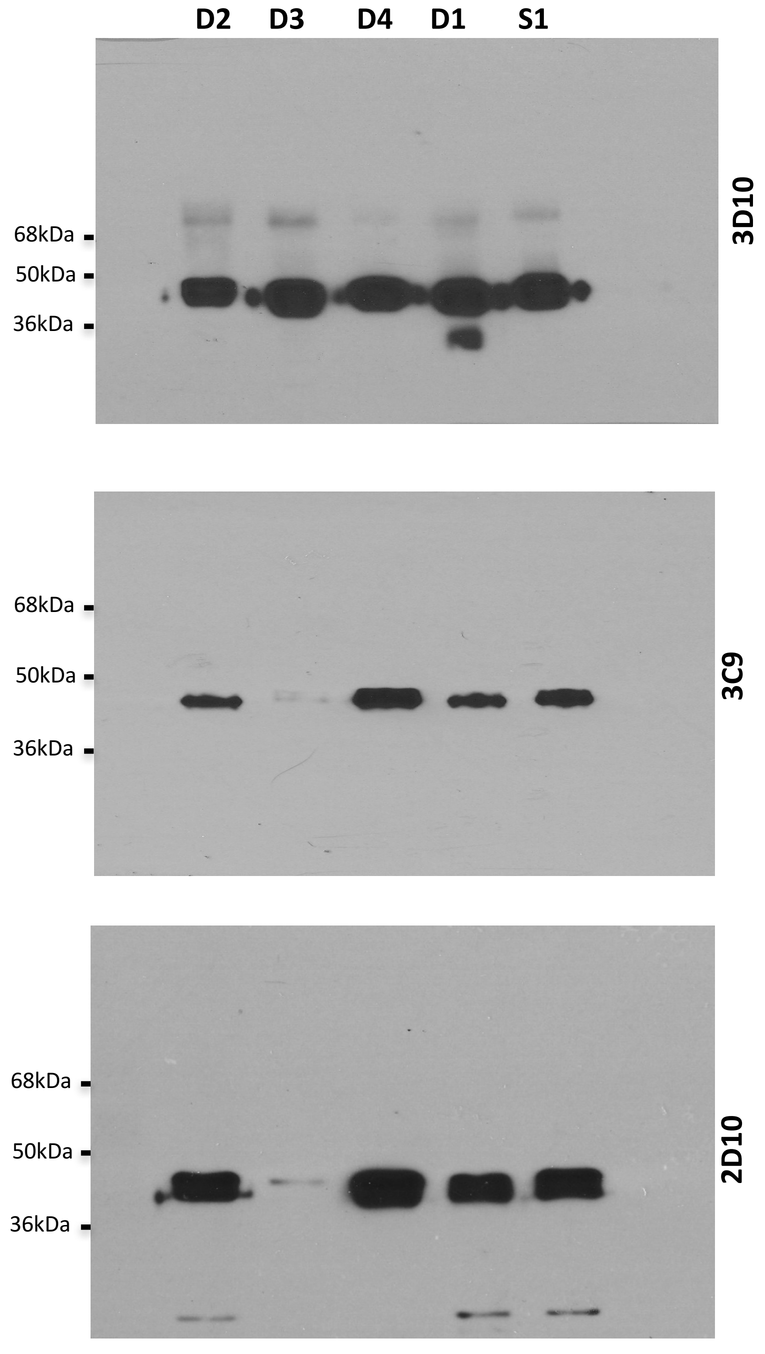

Supplementary Figure S2. Scnned full-length blot showing reactivity of rDEKnull antigens with anti-DBPII conformational dependent (mAbs 3C9 and 2D10) and non-conformational dependent (mAb 3D10) antibodies by Western blot analysis. rSal1 was used as control antigen. DEKnull: D1; DEKnull-2: D2; DEKnull-3: D3; DEKnull-4: D4 and Sal1: S1.

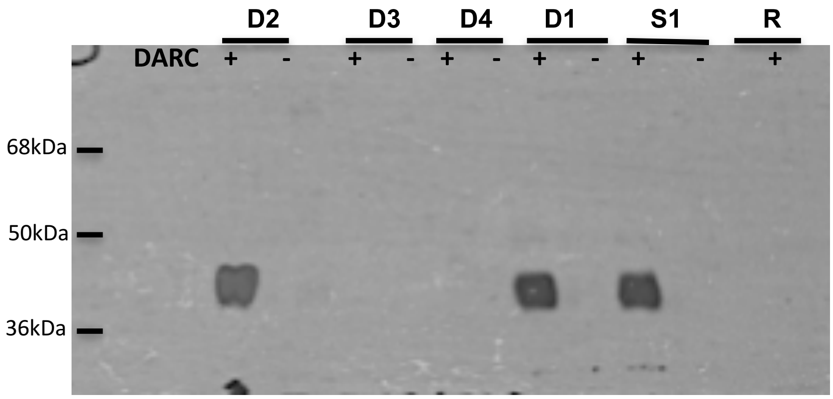

Supplementary Figure S3. Functional analysis of the DEKnull antigens (scanned full-length blot). (**a**) Refolded recombinant DEKnull (D1), DEKnull-2 (D2) and Sal1 (S1) bind to DARC positive (+) but not DARC negative (−) red blood cells, while recombinant DEKnull-3 (D3) and DEKnull-4 (D4) do not bind to either erythrocyte type. DARC positive red blood cells (R) without bound antigen served as control.

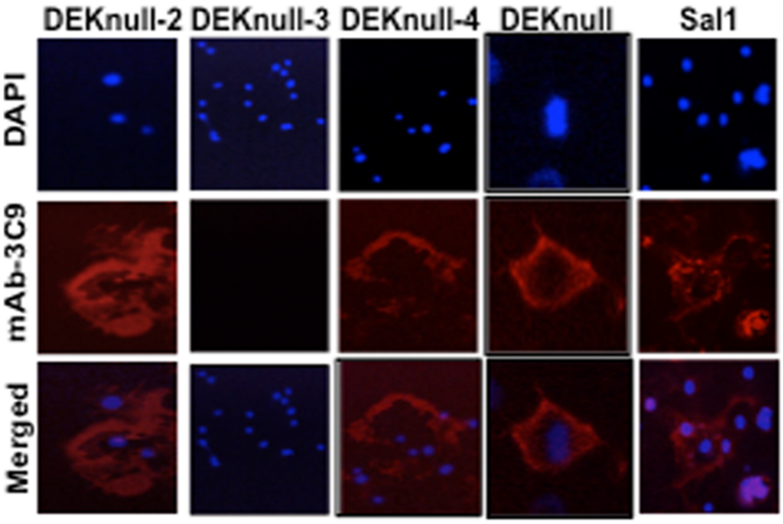

Supplementary Figure S4. Immunofluorescence assay shows cell surface expression of the recombinant DEKnull antigens and native Sal1 on the surface of COS7 cells. The COS7 cell surface-expressed antigens mimics the native protein on the surface of the parasite and avoids the limitations of the recombinant refolded antigens. An anti-DBPII inhibitory antibody, mAb-3C9, was able to detect cell-surface expressed rDEKnull, rDEKnull-2 and rSal1 but not rDEKnull-3 and rDEKnull-4. Thus, confirms that the lack of reactivity of refolded rDEKnull-3 to mAb 3C9 and 2D10 in the ELISA and its lack of binding to DARC were not due to lack of conformation but a simple indication that the mutated residues in DEKnull-3 are important to its function and receptor recognition.

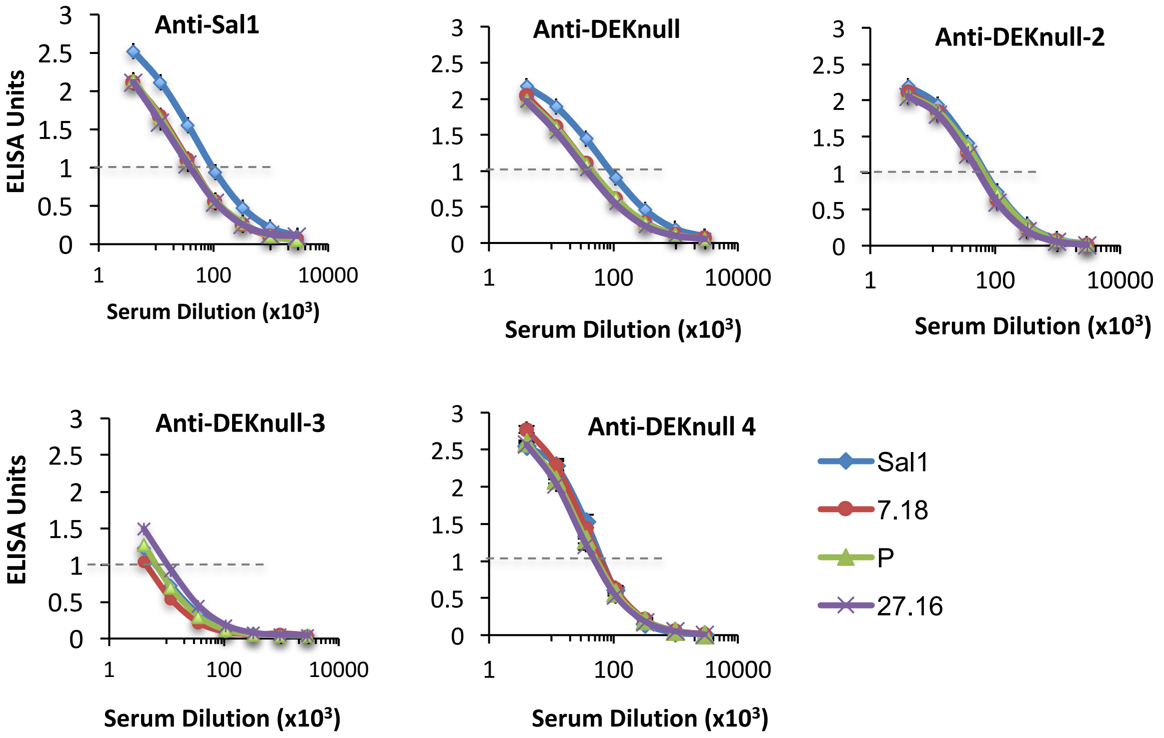

Supplementary Figure S5. Anti-DBPII reactivity profiles. Reactivity of mouse antisera against four naturally occurring variant recombinant DBPII alleles by end-point titration ELISA. Each curve represents a 4-parameter logistic regression for antisera from each mouse (n=15), against the different alleles and error bars represent ±SD. The broken horizontal line represents EU=1.0 used as bases for comparing the reactivity of the different sera

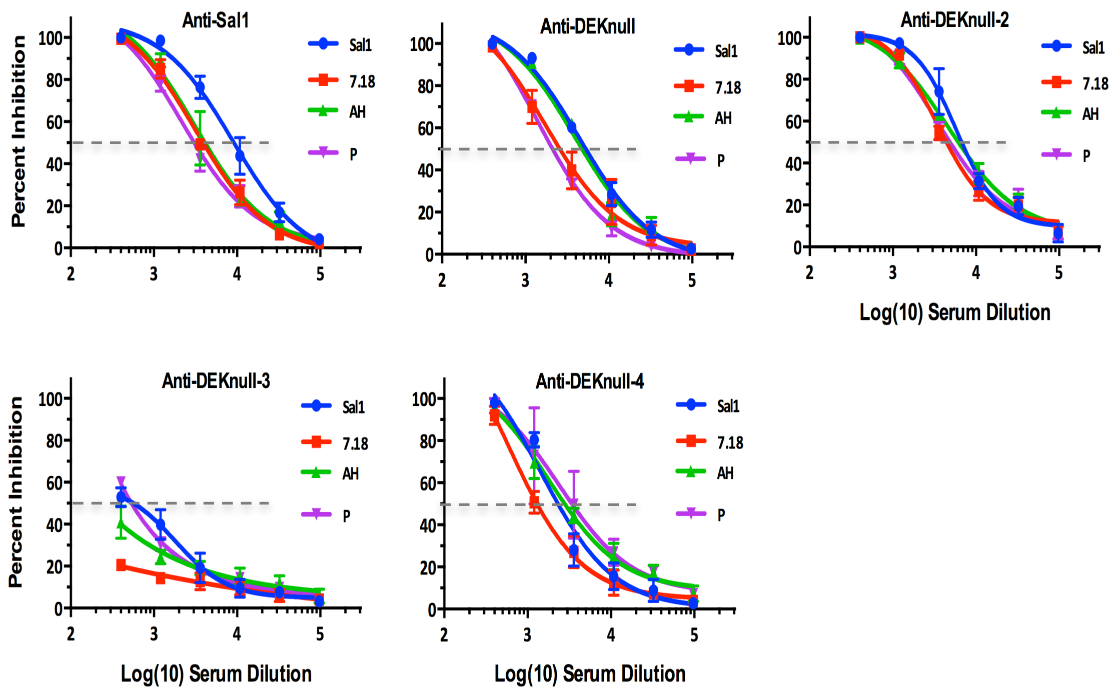

Supplementary Figure S6. Anti-DBPII inhibitory profile. Pooled antisera from against each antigen were tested for inhibition of DBPII binding to DARC on human erythrocytes by the standard COS7 cell assay. Each curve on the charts represents the non-linear regression of two independent experiments, with each dilution tested in triplicate. Error bars represent ± standard deviation. The horizontal broken line indicates the 50% inhibition of binding.

Supplementary Table S1.

Mutated residues in Sal1 to create DEKnull variants

Variant	Amino Acid Residues																											
		299	300	301	302	303	304	305	306	308	312	315	317	318	319	320	321	322	323	327	333	336	337	338	339	340	341	342
**Sal1**	**E**	**V**	**N**	**N**	**T**	**D**	**T**	**N**	**F**	**R**	**F**	**L**	**L**	**K**	**R**	**K**	**L**	**I**	**Y**	**V**	**L**	**N**	**N**	**Y**	**R**	**Y**	**N**	**K**
DEKnull	.	.	.	.	.	.	.	.	.	.	.	.	.	.	.	.	.	.	.	.	.	.	.	.	.	.	.	.
DEKnull-2	.	.	.	.	.	.	.	.	.	A	.	.	.	.	A	.	.	.	.	.	T	.	.	.	.	.	.	.
DEKnull-3		T	A	T	A	T	S	A	A	.	.	.	A	A	.	T	S	A	.	.	.	T	.	.	.	A	S	T
DEKnull-4	A	.	.	.	.	.	.	.	.	.	A	A	.	A	A	.	.	T	A	S	.	A	A	T	A	T	S	A

Variant	Amino Acid Residues																											
	343	344	347	348	349	369	371	374	375	384	385	386	388	389	390	392	393	395	396	397	401	408	409	410	411	412	413	414
**Sal1**	**D**	**F**	**D**	**I**	**R**	**Y**	**K**	**E**	**N**	**D**	**E**	**K**	**Q**	**Q**	**R**	**K**	**Q**	**W**	**N**	**E**	**Q**	**Y**	**S**	**V**	**K**	**K**	**R**	**L**
DEKnull	.	.	.	.	.	.	.	.	.	A	A	T	.	A	T	S	.	T	S	.	.	.	.	.	.	.	.	.
DEKnull-2	.	.	.	.	.	T	A			A	A	T	.	A	.	S	.	.	.	.	.	.	.	.	.	.	.	.
DEKnull-3	.	A	A	A	T	.	.	A	T	.	.	.	.	.	.	.	.	T	A	T	.	A	A	S	T	A	A	S
DEKnull-4	A	T	.	.	.	.	.	.	.	.	.	.	.	.	.	.	.	.	.	.	T	A	.	A	T	A	S	A

Variant	Amino Acid Residues																											
	415	416	417	418	419	420	421	422	424	433	435	436	437	439	440	441	442	444	445	446	447	454	467	492	503	505	511	515
**Sal1**	**K**	**G**	**N**	**F**	**I**	**W**	**I**	**C**	**L**	**Q**	**Y**	**R**	**W**	**R**	**E**	**W**	**G**	**D**	**Y**	**V**	**S**	**Q**	**T**	**K**	**I**	**V**	**V**	**G**
DEKnull	.	.	.	.	.	.	.	.	.	.	.	.	.	.	.	.	.	.	.	.	.	.	.	.	.	.	.	.
DEKnull-2	.	.	T	.	.	.	.	.	A	.	.	.	S	.	.	.	.	.	.	.	A	T	A	A	T	A	A	S
DEKnull-3	.	.	.	A	.	.	S	.	.	T	A	A	.	A	T	S	A	A	T	S	.	.	.	.	.	.	.	.
DEKnull-4	A	T	S	A	A	T	A	A	.	.	.	.	.	.	.	.	.	.	.	.	.	.	.	.	.	.	.	.

Positions of mutated residues in DEKnull variants with reference to the sequence of DBPII-Sal1 (bold) are indicated. Conserved residues are represented by a dot (.).

Table S2. Serology data

ID code	Reactivity Index (ELISA) RI SalI	RI BrzI	RI DEKnull2	BIAb actitivity frequency (COS7	Clusters (Kmeans)
**32**	0.62	1.541	2.425	0	low
**36**	0.69	0.077	0.159	0.1278	low
**54**	0.68	1.286	2.448	0.6333	low
**61**	0.02	0	0.179	0.2812	low
**62**	0.14	0.171	0.559	0.4082	low
**101**	0.18	0.162	2.924	0.01	low
**102**	0.75	0.676	1.531	0.1052	low
**104**	0.09	0.001	0.19	0.2776	low
**125**	0	0.018	0.074	0.1851	low
**189**	3.86	6.921	11.67	0.198	moderate
**204**	0.32	0.128	0.743	0.2633	low
**208**	0.18	0	0.26	0.2159	low
**215**	0.42	0.568	1.129	0.562	low
**216**	0.18	0.196	0.336	0.1851	low
**233**	0.41	1.147	1.676	1	low
**240**	0.09	0	0.299	0	low
**241**	5.93	6.245	14.68	0.0103	moderate
**258**	1.91	2.618	4.42	0.4028	low
**260**	0.43	0.3	1.096	1	low
**262**	7.11	8.771	16.91	0.8328	moderate
**291**	3.94	4.605	9.349	0.9898	moderate
**295**	0.37	0	0.228	0.349	low
**308**	3.98	3.1	8.049	0.74	moderate
**316**	0.07	0	0.178	0	low
**327**	0.26	0.25	0.561	0.9116	low
**328**	0.21	0.184	3.838	0.467	low
**348**	0	0.111	0.85	0.2247	low
**368**	0.02	0.059	0.345	0.1372	low
**405**	0.1	0.132	0.259	0.4608	low
**406**	2.67	2.482	7.931	0.1229	moderate
**411**	0	0.124	0.264	0.3311	low
**412**	2.36	2.453	9.938	0.9892	moderate
**416**	0.65	0.108	0.85	1	low
**422**	0	0.005	0	0	low
**428**	0.31	0.583	0.953	0	low
**459**	0.51	0.3	1.165	0.6089	low
**467**	0.05	0.08	0.385	0.1307	low
**468**	0	0.017	0.057	0	low
**498**	0.22	0.126	0.293	0.0594	low
**504**	1.26	1.001	2.286	0.0615	low

**511**	6.71	10.05	20.92	0.9796	moderate
**516**	6.71	10.91	23.37	0.9659	high
**517**	9.46	1.122	3.491	0.6349	low
**527**	2.44	3.554	9.655	0.8878	moderate
**534**	11.58	14.43	37.38	0.8956	high
**542**	0.04	0.007	0.057	0.3856	low
**547**	0.06	0.464	1.515	0.3252	low
**549**	4.25	6.637	14.06	0.6751	moderate
**550**	0	0.297	0.191	0	low
**553**	1.8	1.684	4.578	0.5813	low
**554**	0.14	0.283	0.335	0	low
**555**	0	0.543	0.339	0.0767	low
**569**	0.18	0.22	0.68	0	low
**580**	0.15	0.057	0.361	0	low
**581**	0.03	0.062	0.049	0	low
**677**	0	0.025	0	0	low
**RP0015**	0.81	2.748	11.4	0.7292	moderate
**RP0016**	1.73	0.464	0.744	0	low
**RP0021**	7.43	16.82	35.9	0.9934	high
**RP0035**	0.96	0.264	0.239	0.07	low
**RP0041**	1.72	0	0.42	0.064	low
**RP0052**	1.08	9.133	11.44	1	moderate
**RP0069**	0.19	0.103	1.067	0.07	low
**RP0072**	0.47	6.778	5.537	0.4108	low
**RP0086**	0.48	4.4	3.232	0.6811	low
**RP0096**	0.507	0.333	0.965	0.331	low
**RP0102**	0	2.557	0.672	0.0016	low
**RP0107**	0.34	0.779	2.034	0.0425	low
**RP0108**	2.23	2.46	2.715	0.5755	low
**RP0136**	0.65	0.144	0.484	0	low
**RP0141**	0	0.124	0.206	0.2411	low
**RP0142**	0	4.484	13.25	0.9527	moderate
**RP0145**	2.45	10.79	28.01	0.9967	high
**RP0148**	0	8.624	19.13	0.9914	moderate
**RP0203**	0.557	0.491	2.42	0.4434	low
**RP0205**	0.47	0.065	0.613	0.1695	low
**RP0209**	0.78	0.703	1.673	0	low
**RP0215**	15.82	9.792	32.84	0.9897	high
**RP0216**	1.54	3.425	12.12	0.9906	moderate
**RP0219**	1.5	0	0.135	0.13	low
**RP0235**	0.447	0.392	0.233	0	low
**RP0236**	0	3.815	3.512	0.4394	low
**RP0251**	1.617	9.609	28.21	0.9917	high
**RP0258**	0.547	1.646	7.568	0	low
**RP0260**	1.69	12.22	33.94	0.9946	high

**RP0262**	0.067	3.837	3.33	0.2243	low
**RP0273**	0.253	0.815	3.735	1	low
**RP0297**	0.493	0.307	0.699	0	low
**RP0302**	0.257	0.238	0.437	0.0182	low
**RP0318**	0	0	0	0.7077	low
**RP0319**	3.743	5.175	15.51	0.9957	moderate
**RP0323**	0.237	0.098	1.491	0.9253	low
**RP0328**	8.01	10.67	31.32	0.9906	high
**RP0330**	0.69	0	0.162	0	low
**RP0350**	0.03	0.005	0.107	0	low
**RP0382**	0.15	0	0	0.1203	low
**RP0388**	1.413	5.678	19.84	0.9906	moderate
**RP0402**	0.53	0.765	0.463	0.094	low
**RP0420**	0	0.077	0.331	0.106	low
**RP0469**	0.22	0	0.047	0.168	low
**RP0471**	0.13	0	0	0	low
**RP0482**	1.42	1.471	1.396	0	low
**RP0517**	0.68	0	1.005	0.339	low
**RP0519**	1.16	0.255	1.488	0.1578	low
**RP0530**	0.45	0	0.221	0	low
**RP0532**	0.05	0	0	0.118	low
**RP0543**	1.037	0	0.01	0	low
**RP0545**	0.69	1.328	3.601	0	low
**RP0547**	1.19	0.189	0	0.3753	low
**RP0556**	0.55	1.099	4.253	0.679	low
**RP0571**	0.28	1.39	0.444	0.869	low
**RP0589**	0.03	0.324	2.235	1	low
**RP101B**	0.96	0.12	1.438	0.034	low
**RP101C**	1.08	0.945	2.671	0.064	low
**RP102B**	0.8	0	0.798	0.0104	low
**RP102C**	0.67	0.557	0.837	0	low
**RP104B**	0.26	0	0.299	0.1823	low
**RP104C**	0.18	0.085	0.589	0.0043	low
**RP125B**	0.05	0.022	0	0	low
**RP125C**	0.17	0.213	0	0	low
**RP189B**	2.07	0.156	1.565	0.657	low
**RP189C**	1.7	0.498	2.793	0.4792	low
**RP204B**	1.29	0.05	0.778	0.9437	low
**RP204C**	2.1	0.277	1.744	0.182	low
**RP208B**	0.07	0	0.059	0.064	low
**RP208C**	0.42	0.411	0	0	low
**RP215B**	2.55	1.083	2.768	0.1899	low
**RP215C**	1.05	1.326	2.51	0	low
**RP216B**	0.36	0.246	0	0	low
**RP216C**	0.96	0.47	0.621	0.0135	low

**RP233**	4.1	2.021	11.13	0.934	moderate
**RP233C**	6.44	3.128	7.223	0.9169	moderate
**RP240**	0.73	0.057	1.168	0.0051	low
**RP240C**	0.77	0.446	1	0	low
**RP241**	2.13	0.741	1.545	0.5922	low
**RP241C**	1.28	0.798	2.37	0	low
**RP258**	10.1	10.91	32.57	0.991	high
**RP258C**	15.7	10.26	22.01	0.9864	high
**RP260**	3.86	0.627	2.069	0.8222	low
**RP260C**	2.51	0.595	2.971	0.5684	low
**RP262**	5.87	5.389	15.97	0.9967	moderate
**RP262C**	12.8	6.201	14.92	0.67	moderate
**RP291**	7.11	4.109	16.87	0.9978	moderate
**RP291C**	14.5	5.917	14.7	0.9956	moderate
**RP295**	0.69	0.044	0.549	0.174	low
**RP295C**	0.32	0.181	0.479	0.0368	low
**RP308**	6.53	4.978	14.61	0.9984	moderate
**RP308C**	11.7	5.893	11.47	0.9918	moderate
**RP316**	0.55	0.062	0.617	0.2319	low
**RP316C**	0.94	0.329	0.561	0.3182	low
**RP327**	2.33	0.052	0.513	0.9653	low
**RP327C**	3.05	0.194	1.499	0.2683	low
**RP328**	0.94	0.197	0.801	0.3126	low
**RP328C**	0.5	0.388	1.769	0.4403	low
**RP32**	3.49	1.567	5.585	0.478	low
**RP32C**	9.58	5.193	8.527	0.3409	moderate
**RP348**	0.23	0.016	0.387	0	low
**RP348C**	0.26	0.222	0.31	0	low
**RP368**	5.56	0	0	0.1753	low
**RP368C**	0.74	0.073	0	0.0587	low
**RP36**	0.76	0.126	1.39	0.18	low
**RP36C**	0.13	0.246	1.489	0.12	low
**RP405**	0.98	0.9	0.316	0.43	low
**RP405C**	1.27	1.74	0.45	0.42	low
**RP406**	2.53	1.008	3.571	0.8229	low
**RP406C**	1.7	0.96	4.083	0.0776	low
**RP411**	0.22	0	0.168	0.099	low
**RP411C**	0.24	0.37	0.865	0	low
**RP412**	0.46	0.186	0.155	0	low
**RP412C**	0.33	0.352	0.56	0.0041	low
**RP416**	1.57	0.023	1.59	0.7257	low
**RP416C**	4.03	0.812	2.446	1	low
**RP422**	0.12	0	0.179	0.2555	low
**RP422C**	0.19	0.166	0.495	0	low
**RP428**	1.8	1.358	1.988	0	low

**RP428C**	1.43	1.616	2.057	0	low
**RP459**	1.27	0.251	1.888	0.3933	low
**RP459C**	2.11	1.176	1.674	0.3577	low
**RP467**	0.55	0	1.126	0.6695	low
**RP467C**	0.32	0.144	1.566	0.3072	low
**RP468**	0.15	0	0.014	0	low
**RP468C**	0.04	0.164	0.696	0.3359	low
**RP498B**	0.41	0.532	0.733	0.1615	low
**RP498C**	0.68	0.652	1.167	0.027	low
**RP504B**	3.41	1.51	6.066	0.5761	low
**RP504C**	11.5	3.24	13.16	0.9897	moderate
**RP511B**	10.8	26.18	32.75	0.9967	high
**RP511C**	18.7	19.13	31.4	0.99	high
**RP516B**	12.1	24.02	32.48	0.9962	high
**RP516C**	20.1	18.38	32.82	0.9981	high
**RP517B**	4.43	7.013	10.74	0.873	moderate
**RP517C**	5.04	4.199	9.574	0.8084	moderate
**RP527B**	9.52	10.36	26.23	0.8596	high
**RP527C**	12.2	10.98	17.86	0.8261	moderate
**RP534B**	12.1	20.3	31.73	0.9934	high
**RP534C**	22	19.06	31.8	0.9938	high
**RP542B**	0.19	0.089	0.206	0.0768	low
**RP542C**	0.37	0.175	0.198	0.0379	low
**RP547B**	0.53	0.65	1.494	0	low
**RP547C**	0.87	0.506	1.038	0	low
**RP549B**	4.58	6.593	12.76	0.9938	moderate
**RP549C**	6.76	5.605	11.22	0.9907	moderate
**RP54B**	5.76	4.933	11.41	1	moderate
**RP54C**	5.67	4.146	7.345	1	moderate
**RP550B**	0.39	0.215	0.268	0.0202	low
**RP550C**	0.58	0.202	0.117	0.0543	low
**RP553B**	2.55	2.55	3.349	0.5164	low
**RP553C**	2.93	2.81	2.966	0.121	low
**RP554B**	0.47	0.266	1.979	0	low
**RP554C**	0.75	0.413	1.948	0.3782	low
**RP555B**	0.34	0	0.133	0.5426	low
**RP555C**	0.39	0.158	0.415	0.4676	low
**RP569**	2.06	2.42	1.677	0.0673	low
**RP569C**	1.77	2.02	0.97	0.092	low
**RP580**	0.18	0.086	0.179	0	low
**RP580C**	0.27	0.173	0.339	0	low
**RP581**	0.11	0.041	0.452	0.21	low
**RP581C**	0.13	0.089	0.126	0.187	low
**RP61**	5.05	1.955	4.788	0.124	low
**RP61C**	0.94	0.383	0.821	0	low

**RP62**	0.09	0	0.344	0	low
**RP62C**	0.11	0.039	0.287	0	low
**RP677**	0.19	0.228	0.649	0	low
**RP677C**	0.06	0.068	0.143	0	low

ID samples in each survey			
**baseline**	**6 month**	**12 month**	**6 year**
RP0004	RP544B	RP544C	.
RP0005	RP543B	RP543C	.
RP0007	RP571B	RP571C	.
RP0015	RP504B	RP504C	RP504
RP0016	RP498B	RP498C	RP505
RP0018	RP513B	RP513C	RP506
RP0020	RP512B	RP512C	RP507
RP0021	RP511B	RP511C	RP508
RP0023	RP514B	RP514C	RP509
RP0029	RP88B	RP88C	RP510
RP0035	RP62B	RP62C	RP511
RP0041	RP61B	RP61C	RP512
RP0052	RP54B	RP54C	RP513
RP0054	RP55B	RP55C	RP514
RP0069	RP36B	RP36C	RP515
RP0072	RP32B	RP32C	RP516
RP0076	RP31B	RP31C	RP517
RP0086	RP569B	RP569C	RP518
RP0096	RP328B	RP328C	RP519
RP0101	RP179B	RP179C	RP520
RP0102	RP547B	RP547C	RP521
RP0107	RP554B	RP554C	RP522
RP0108	RP553B	RP553C	RP523
RP0111	RP615B	RP615C	RP524
RP0118	RP548B	RP548C	RP525
RP0121	RP546B	RP546C	RP526
RP0136	RP411B	RP411C	RP527
RP0141	RP550B	RP550C	RP528
RP0142	RP549B	RP549C	RP529
RP0145	RP258B	RP258C	RP530
RP0148	RP527B	RP527C	RP531
RP0149	RP759B	RP759C	RP532
RP0203	RP189B	RP189C	RP533
RP0205	RP468B	RP468C	RP534
RP0209	RP204B	RP204C	RP535
RP0210	RP210B	RP210C	RP536
RP0215	RP516B	RP516C	RP537
RP0216	RP517B	RP517C	RP538
RP0219	RP581B	RP581C	RP539
RP0235	RP216B	RP216C	RP540
RP0236	RP215B	RP215C	RP541
RP0247	RP317B	RP317C	RP542
RP0251	RP233B	RP233C	RP543

RP0254	RP236B	RP236C	RP544
RP0258	RP241B	RP241C	RP545
RP0260	RP262B	RP262C	RP546
RP0262	RP240B	RP240C	RP547
RP0268	RP245B	RP245C	RP548
RP0273	RP260B	RP260C	RP549
RP0280	RP267B	RP267C	RP550
RP0289	RP265B	RP265C	RP551
RP0290	RP277B	RP277C	RP552
RP0297	RP295B	RP295C	RP553
RP0302	RP316B	RP316C	RP554
RP0304	RP321B	RP321C	RP555
RP0308	RP304B	RP304C	RP556
RP0316	RP597B	RP597C	RP557
RP0318	RP555B	RP555C	RP558
RP0319	RP308B	RP308C	RP559
RP0323	RP327B	RP327C	RP560
RP0328	RP534B	RP534C	RP561
RP0330	RP542B	RP542C	RP562
RP0338	RP340B	RP340C	RP563
RP0350	RP348B	RP348C	RP564
RP0369	RP365B	RP365C	RP565
RP0381	RP386B	RP386C	RP566
RP0382	RP368B	RP368C	RP567
RP0388	RP291B	RP291C	RP568
RP0396	RP624B	RP624C	RP569
RP0402	RP102B	RP102C	RP570
RP0410	RP182B	RP182C	RP571
RP0412	RP285B	RP285C	RP572
RP0420	RP104B	RP104C	RP573
RP0421	RP80B	RP80C	RP574
RP0437	RP154B	RP154C	RP575
RP0469	RP677B	RP677C	RP576
RP0471	RP125B	RP125C	RP577
RP0482	RP101B	RP101C	RP578
RP0490	RP114B	RP114C	RP579
RP0494	RP98B	RP98C	RP580
RP0517	RP467B	RP467C	RP581
RP0519	RP459B	RP459C	RP582
RP0529	RP442B	RP442C	RP583
RP0530	RP580B	RP580C	RP584
RP0532	RP422B	RP422C	RP585
RP0543	RP208B	RP208C	RP586
RP0545	RP428B	RP428C	RP587
RP0547	RP412B	RP412C	RP588

RP0550	RP413B	RP413C	RP589
RP0556	RP406B	RP406C	RP590
RP0561	RP398B	RP398C	RP591
RP0569	RP315B	RP315C	RP592
RP0571	RP405B	RP405C	RP593
RP0577	RP462B	RP462C	RP594
RP0589	RP416B	RP416C	RP595
RP0707	RP379B	RP379C	RP596

Table S2: Serological_data: Reactivity Indexes (ELISA) of Sal-1, Brz-1 and DEKnull-2 and BIAbs activity frequency (COS binding-inhibition assay) of each sample from individuals in the malaria endemic area of the Brazilian Amazon. K-means analysis shows samples grouped into Low, moderate or high reactive groups based on reactive index (ELISA) and binding inhibitory activity (COS7 assay).
